# Effects of N3SA Analogues on Cerebral and Peripheral Arteriolar Vasomotion in Spontaneously Hypertensive Rats

**DOI:** 10.3390/ijms27021006

**Published:** 2026-01-20

**Authors:** Dominga Lapi, Giuseppe Federighi, Maria Paola Tramonti Fantozzi, Gianpiero Garau, Rossana Scuri

**Affiliations:** 1 Department of Biology, University of Pisa, 56127 Pisa, Italy; dominga.lapi@unipi.it; 2 Department of Translational Medicine and New Technologies in Medicine and Surgery, University of Pisa, 56127 Pisa, Italy; mariapaola.fantozzi@med.unipi.it (M.P.T.F.); rossana.scuri@unipi.it (R.S.); 3 BioStructures Lab, Istituto Italiano di Tecnologia (IIT@NEST), 56127 Pisa, Italy; gianpierogarau@gmail.com; 4 Laboratori ALIVEDA, 56042 Pisa, Italy

**Keywords:** arteriolar diameter changes, femoral microcirculation, pial microcirculation, power spectrum analysis, thiazides

## Abstract

Thiazides are among the most efficacious and commonly used drugs for the treatment of hypertension. The nanomolar stabilizer N3SA binds specifically to the recently discovered thiazide-binding site of the membrane target NAPE-PLD, showing sustained arterial blood pressure-lowering effects and vasodilation in spontaneous hypertensive rats (SHRs). To further support the relation between stabilizers anchored to NAPE-PLD and their beneficial effects on hypertension, we selected compound analogues of N3SA with chemical modifications at the three target-interacting sulfonic groups, including the drug Suramin. Each compound was injected i.v in an adult SHR (systolic blood pressure of 217 ± 5 mmHg) to evaluate the frequency components contribution to cerebral and peripheral arteriolar vasomotion. We visualized the pial and rectus femoral muscle microcirculation by Epi-illumination, measuring changes in the rhythmic arteriolar diameter. Findings showed that the minor structural differences in compounds correlated with the contribution of the six different frequency components affecting the arterial tone, as well as their vasodilatory effects, in both cerebral and femoral muscle arterioles. These results provide evidence that the spectra analysis of the regulation mechanisms of vascular tone and arterial blood pressure can accurately reflect the structure–activity correlations of different analogues of an antihypertensive compound.

## 1. Introduction

Thiazide diuretics (TZDs) are among the most frequently prescribed antihypertensive medications and are also widely used in the management of heart failure and stroke [1]. It is generally accepted that these drugs exert their long-term antihypertensive action primarily through a reduction in total peripheral vascular resistance [2,3,4]. However, despite their clinical utility, the downstream cellular and molecular pathways mediating their effects, as well as how they modulate vascular permeability, have remained mysterious for decades [5]. Their unique chronic blood pressure-lowering effects are only now being fully elucidated.

After the identification of the sodium chloride cotransporter (NCC) as a possible renal target, several hypotheses proposed the existence of at least one extrarenal target to rationalize their vascular effects and help account for their robust blood pressure-lowering effects [6], ranging from indirect modulation of upstream neurohumoral systems to a direct inhibitory effect on vascular tone regulation [7,8,9,10]. Nevertheless, a clear consensus on the identity and functional significance of such an extrarenal target was not reached.

**Figure 1:**
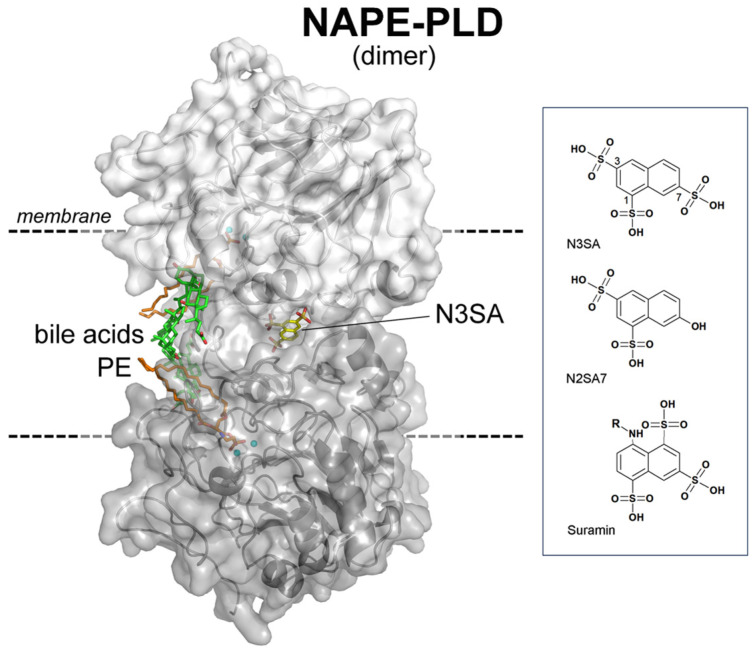
**Structure of the human membrane NAPE-PLD in a complex with N3SA.** View of the overall homodimeric architecture of NAPE-PLD, showing its ~9 Å-wide internal channel located at the interface of protein subunits (in light and dark grey). Bound to the active site is a molecule of phosphatidylethanolamine (PE, carbons in orange), which indicates how the substrate NAPE (N acylphosphatidylethanolamines) goes in and binds the binuclear zinc-containing active site (zinc ions in cyan). The bound molecule of N3SA (carbons in yellow) is located at the channel rim and acts as an allosteric stabilizer, reinforcing the homodimer stabilization mediated by bile acid molecules (carbons in green). Bile acids mediate the association of NAPE-PLD with membrane phospholipids. The box on the right shows the chemical structure of N3SA and selected analogues.

Recent structural and functional findings demonstrated that the membrane N-acyl phosphatidylethanolamine phospholipase D (NAPE-PLD) of the endocannabinoid system (ECS) has the potential to be a previously unrecognized extrarenal mediator of the cardiovascular effects of TZDs [11,12]. The compound naphthalene (1,3,7) trisulfonic acid (N3SA) was identified as a potent stabilizer of NAPE-PLD (~33 nM), which specifically binds to the thiazide binding site located within its internal dimeric channel (Figure 1).

Low-dose administration of N3SA (0.1–0.2 mg/kg/day) in spontaneously hypertensive rats (SHRs) resulted in insignificant and sustained reductions in arterial blood pressure and vascular resistance, supporting the clinical relevance of NAPE-PLD as a novel therapeutic target for hypertension [11]. The beneficial effects observed with the compound N3SA in the animal model and the fact that the enzyme is a key player of the ECS suggested that the protein may be exploited for stress adaptation and neurovascular disorders [11,12,13]. In line with this, studies on rodent knockouts and on genetic variants detected in dog breeds showed dysfunctions of the target associated with metabolic and myelin diseases [14,15]. Ongoing research efforts are currently focused on optimizing the pharmacological stabilization of NAPE-PLD to enhance therapeutic efficacy while minimizing side effects commonly associated with TZD therapy, such as metabolic disturbances and hyponatremia. Building upon these findings, the present study aimed to investigate the structure–activity relationships of N3SA by assessing its effects on arteriolar vasomotion in both cerebral and peripheral microcirculation. To this end, we selected analogues bearing targeted modifications at the three sulfonic acid groups known to interact with the internal binding channel of NAPE-PLD. Among the compounds, we tested the drug Suramin, used for Trypanosoma infections [16].

Here, we visualized the microvascular networks in pial (cerebral) and femoral muscle (peripheral) circulations to monitor real-time changes in arteriolar diameter after the administration of N3SA and its analogues, quantifying the contribution of six distinct frequency components associated with vasomotor activity.

The first interpretation of this approach was proposed by Stefanovska et al. [17]. In their work, they showed that wavelet-based time-frequency analysis of laser Doppler flowmetry signals, obtained by placing the probe on the skin, could identify five distinct frequency components. These were associated with specific physiological mechanisms: cardiac activity (0.6–2 Hz), respiration (0.15–0.6 Hz), myogenic activity of the vascular smooth muscle (0.052–0.15 Hz), sympathetic nervous system activity (0.021–0.052 Hz), and very low-frequency oscillations related to endothelial function (0.0095–0.021 Hz). The latter component was found to be modulated by the endothelium-dependent vasodilator acetylcholine [17].

Subsequently, our group observed that the frequency component related to endothelial activity could be mainly attributed to two distinct mechanisms in cerebral arterioles: one NO-independent, associated with the endothelium-derived hyperpolarizing factor (EDHF), and the other NO-dependent, linked to nitric oxide (NO) release [18]. As a result, we identified a total of six frequency components that can monitor the vasomotor activity. Here, we demonstrate that our methodology can discriminate even subtle structural differences among analogues of a chemical probe, which differentially influence the frequency-dependent mechanisms governing vascular tone and blood pressure regulation. The aim of the present study is to establish a correlation between the chemical architecture of N3SA-derived compounds and their vasodilatory effects on the membrane target NAPE-PLD, as reflected by specific oscillatory patterns in the microvasculature. Integrating high-resolution vascular imaging with frequency-domain analysis, we also provide a mechanistic framework useful to investigate how small chemical modifications can affect the pharmacodynamic profile of an antihypertensive agent.

## 2. Results

### 2.1. Effects of N3SA Analogues on Vascular Tone in Cerebral Microcirculation

**Figure 2:**
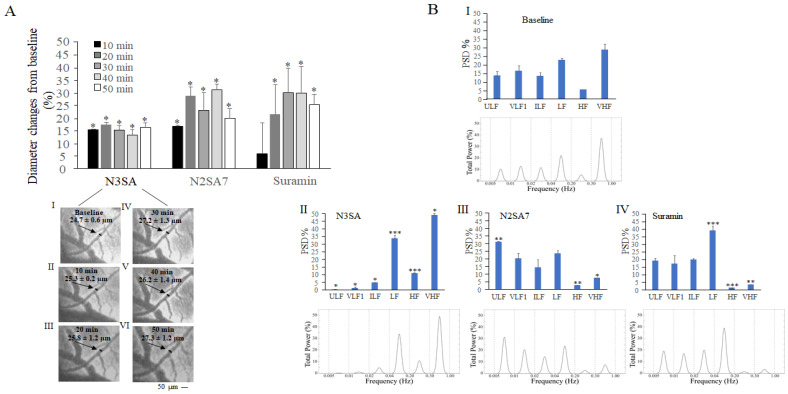
**Effects on vascular tone in cerebral microcirculation.** (**A**) The time course of the percent diameter changes from the baseline was taken as 100%. Below are images of an order 2 arteriole in the parietal pial microvasculature of a SHR i.v. injected with N3SA captured in basal conditions (**I**) and 10 (**II**), 20 (**III**), 30 (**IV**), 40 (**V**), and 50 min (**VI**) after the drug administration (see Supplementary Materials). Values indicate the computer-assisted calculated arteriolar diameter. (**B**) Total Power Distribution Across Six Frequency Bands: Power Spectral Density (PSD) Analysis. The PSD was estimated over the 0–4.5 Hz range. To specifically assess the relative distribution of power within the 0.005–1 Hz band of interest, the PSD was normalized to the total integrated power of that range. The frequency components expressed as percent normalized power spectral density (PSD) plotted were calculated under basal conditions (**I**), 20–40 min after N3SA administration (**II**) (*n* = 3), N2SA7 administration (**III**) (*n* = 3), and Suramin administration (**IV**) (*n* = 3). The subplots illustrate the cumulative power (expressed as a percentage) obtained by integrating the normalized PSD across the following six frequency sub-bands: ULF (0.005–0.01 Hz), VLF1 (0.01–0.02 Hz), VLF2 (0.02–0.04 Hz), LF (0.04–0.20 Hz), HF (0.20–0.30 Hz) and VHF (0.30–1.00 Hz). All reported values are mean ± SE. Statistical significance was set at *p* < 0.05. Asterisks indicate significant statistical differences compared with baseline. * *p* < 0.05, ** *p* < 0.01, *** *p* < 0.0001.

All three pharmacological agents, N3SA, N2SA7, and Suramin (see Section 4), were tested on pial arterioles in the parietal area under basal conditions (baseline) and every 10 min starting from 10 min up to 50 min post intravenous administration. The pial arterioles showed a significant vasodilatory response with different patterns among the treatment groups (Figure 2A), always perduring the whole observation period. In SHRs treated with N3SA the diameter of order 2 arterioles changed from 24.7 ± 0.6 µm (baseline) to 27.0 ± 1.0 µm at 50 min, in SHRs treated with N2SA7 from 21.2 ± 0.5 µm (baseline) to 25.3 ± 0.4 µm at 50 min and in SHRs treated with Suramin from 25.7 ± 1.6 µm (baseline) to 32.0 ± 0.9 µm at 50 min.

In the time interval between 20 and 40 min after compound administration, the rhythmic diameter changes in order 2 arterioles were measured to perform a spectral analysis. The different agents modulated the frequency components of vascular oscillations in distinct ways. N3SA led to a significant reduction in the oscillatory components associated with endothelial function (ULF and VLF1) and neurogenic activity (ILF) (Figure 2B(I,II)), simultaneously enhancing the component linked to myogenic activity (LF) compared with baseline conditions. On the other hand, there was a significant increase in frequency components associated with respiration and heart rates (HF and VHF) compared with baseline conditions. N2SA7 (Figure 2B(I,III)) significantly enhanced ULF without affecting VLF1, ILF, and LF frequency components, while HF and VHF were significantly reduced. Suramin (Figure 2B(I,IV)) did not affect ULF, VLF1, and ILF components, but determined a significant increase in the LF component correlated to myogenic activity compared with baseline conditions. HF and VHF components were consequently reduced.

### 2.2. Effects of N3SA Analogues on Vascular Tone in Femoral Muscle Microcirculation

**Figure 3:**
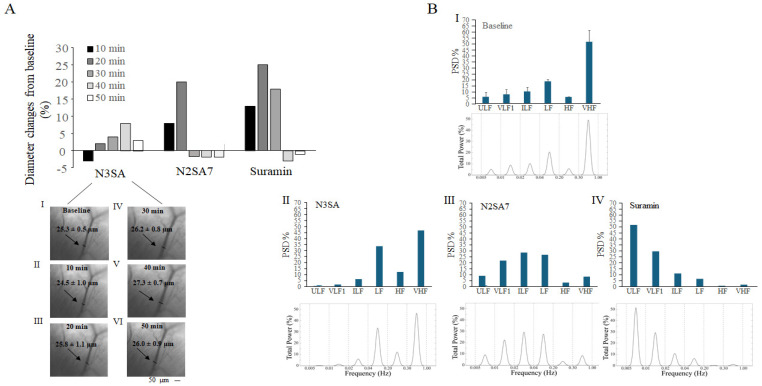
**Effects on vascular tone in femoral muscle microcirculation.** (**A**) The time course of the percent diameter changes from the baseline was taken as 100%. Below are images of an arteriole whose diameter was like that of an order 2 pial arteriole in the femoral muscle microvasculature of one SHR i.v. injected with N3SA acquired in basal conditions (**I**) and 10 (**II**), 20 (**III**), 30 (**IV**), 40 (**V**), and 50 min (**VI**) after the drug administration (see Supplementary Materials). Values indicate the computer-assisted calculated arteriolar diameter. (**B**) Total Power Distribution Across Six Frequency Bands: Power Spectral Density (PSD) Analysis. The PSD was estimated over the 0–4.5 Hz range. To specifically assess the relative distribution of power within the 0.005–1 Hz band of interest, the PSD was normalized to the total integrated power of that range. The frequency components expressed as percent normalized power spectral density (PSD) plotted were calculated in basal conditions (**I**), 20–40 min after N3SA administration (**II**), 10–30 min after N2SA7 administration (**III**), and 10–30 min after Suramin administration (**IV**).

In Figure 3, the effects of N3SA, N2SA7, and Suramin are described. On femoral muscle arterioles, the three compounds produced different actions: N3SA initially induced vasoconstriction (24.5 ± 1.0 µm compared to baseline value of 25.3 ± 0.5 µm) and further vasodilation starting from 20 min up to 50 min with the most pronounced difference in the effect observed at 40 min (27.3 ± 0.7 µm, Figure 3A). Both N2SA7 and Suramin caused a greater vasodilation in comparison with N3SA with the nadir of the effects at 20 min (N2SA7: 31.7 ± 0.9 µm compared to baseline value of 26.5 ± 0.6 µm; Suramin: 36.1 ± 0.5 µm with respect to baseline of 28.8 ± 0.8 µm), followed by vasoconstriction (Figure 3A).

The power spectrum analysis we used allowed us to identify the same six distinct rhythmic diameter oscillations observed in pial arterioles, which were affected by the three compounds analyzed (Figure 3B). The data were obtained by measuring the rhythmic diameter changes in a time interval of 20 min, when the arterioles exhibited vasodilation. N3SA treatment resulted in a pronounced reduction in the frequency components associated with endothelial and neurogenic activity (ULF, VLF1, and ILF), while promoting an increase in the myogenic (LF) and respiratory-related (HF) components. The very high-frequency (VHF) component remained unaffected (Figure 3B(I,II)). In contrast, N2SA7 induced an overall enhancement of low-frequency components linked to endothelial and neurogenic regulation (ULF, VLF1, and ILF), as well as the myogenic component (LF), while concurrently decreasing the HF and VHF components (Figure 3B(I,III)). Suramin caused a marked increase in the endothelial-related components (ULF and VLF1), did not affect the neurogenic activity-related component (ILF), and caused a reduction in the myogenic, respiratory, and cardiac components (LF, HF, and VHF, respectively), (Figure 3B(I,IV)).

## 3. Discussion

In the present study, we provide the first evidence that N3SA and a selection of its structural analogues (N2SA7 and Suramin) exert significant vasodilatory effects in both the cerebral (pial) and peripheral (femoral) microcirculation of SHR. Our results show that their action relates with their structural differences. Previously, we reported that N3SA binds into the same site of NAPE-PLD as the antihypertensive drug hydrochlorothiazide and showed that this binding promotes a reduction in arterial blood pressure in SHR [11]. This finding offered a mechanistic explanation for the acute and chronic antihypertensive effects of thiazide diuretics mediated by NAPE-PLD at the systemic level, beyond the renal NCC inhibition [8,19,20,21,22].

Here, time course analysis of the vascular responses revealed significant differences in vasodilatory outlines among the three compounds. N3SA induced continuing vasodilation in both vascular districts, lasting up to 50 min after intravenous administration. In contrast, N2SA7 and Suramin elicited a continuing vasodilation in cerebral microcirculation and a more pronounced yet transient vasodilation, which diminished significantly by 30–40 min post-administration in femoral microcirculation. These observations suggest a specificity of action related to the small chemical differences among compounds, which can affect their interactions with NAPE-PLD or eventually alternative off-targets.

To explore the underlying mechanisms, we performed power spectral analysis of the different frequency oscillations in pial and femoral arterioles. Under baseline conditions, the six distinct frequency components yet identified in pial arterioles [18] corresponding to endothelial (NO-independent and NO-dependent), neurogenic, myogenic, respiratory, and cardiac influences on vascular tone have also been identified in femoral arterioles, for the first time.

In pial parietal arterioles, we observed that N3SA caused a significant reduction in endothelial (ULF, VLF1) and neurogenic (ILF) oscillations, alongside a significant increase in myogenic (LF), respiratory (HF), and cardiac (VHF) activity. This variation suggests a suppressive effect on local regulatory mechanisms, consistent with an inhibition of vasoactive lipid signalling or a modulation of sympathetic tone via endocannabinoid pathways [22,23,24].

In the same district, on the contrary, N2SA7 significantly enhanced the NO-independent endothelial component and significantly decreased the respiratory (HF) and cardiac (VHF) components, indicating a pronounced stimulatory effect on local vascular control, potentially reflecting accessory mechanisms or alternative targets. Suramin determined a significant increase in myogenic activity (LF) and reduced the respiratory (HF) and cardiac (VHF) activity, suggesting a marked control on the vascular smooth muscle cells.

In femoral arterioles, N3SA reduced endothelial and neurogenic oscillations and enhanced myogenic, respiratory, and cardiac components, as we observed in pial arterioles. On the contrary, N2SA7 and Suramin induced different effects on the femoral arteriolar tone in comparison with those observed in pial vessels. Specifically, in the femoral district N2SA7 increased the NO-dependent endothelial components as well as the neurogenic and myogenic components, reducing the other components. The administration of Suramin determined a marked enhancement of the endothelial components and a marked reduction in the myogenic, respiratory, and cardiac components in the femoral district.

When interpreting our data, it should be considered that the six frequency components of the spectrum are related to each other, and in our analysis, they are illustrated as percentages in the histograms. As a result, the variation in one of the components modulates all others, and the graph illustrates those that are predominant. The vascular tone of the cerebral microcirculation showed initially a nearly homogenous distribution of the six different frequency components, which include the endothelial (ULF and VLF1) and neurogenic (ILF) oscillations, as well as the myogenic (LF), respiratory (HF), and cardiac (VHF) activity (Figure 2). On the contrary, the cardiac (VHF) component prevails in the femoral muscle microcirculation (Figure 3B).

Another important aspect to consider when interpreting our data is that the spectral analysis was carried out in a time period of almost 20 min. This time was essential to monitor the variation in all components appreciably, including those at low frequency. During this time, the vessels dilated due to the administration of the compounds. We chose a time frame in which the diameter of the arterioles tended to increase and not decrease, as all three drugs tested showed hypotensive effects [11] and induced vasodilation. Moreover, the vasomotility lapsed during vessel constriction [25,26,27,28].

Taken together, our data demonstrate that all three NAPE-PLD ligands promote vasodilation. At the same time, they shed light on their mechanism of hypertension reduction. They show slightly different effects and temporal profiles at central and peripheral microcirculations. N3SA appears to favour a stable, suppressive effect on endothelial and neurogenic modulation, consistent with sustained blood pressure control. N2SA7 and Suramin act more acutely, showing short-term effects. With respect to N3SA, these analogues essentially differ in the absence of a sulfonic acid group in position (7) or (3) of the naphthalene ring, respectively (Figure 1). Singularly, these positions are critical for absorption, distribution, residence time, and the effect of the compound. Previously, we showed that the presence of a sulfonic acid group in position (1) was essential for the antihypertensive effect of N3SA [11].

## 4. Materials and Methods

All experimental procedures involving animals were conducted in strict accordance with the Guide for the Care and Use of Laboratory Animals published by the U.S. National Institutes of Health. The study protocol received prior approval from the Ethics Committee for Animal Experimentation of the University of Pisa and from the Italian Ministry of Health (Authorization No. 156/2017-PR, approval date 13 February 2017).

### 4.1. Experimental Groups

The experimental procedures were conducted on a total of twelve adult spontaneously hypertensive rats (SHRs), with a basal systolic blood pressure of 217 ± 5 mmHg measured using the rat tail cuff method with the MRBP Blood Pressure System (IITC Life Science Inc., Los Angeles, CA, USA). Animals were randomly divided into three experimental groups (*n* = 4 for each group), each receiving a different pharmacological treatment administered intravenously at a dose of 0.1 mg/kg of body weight over a period of 5 min.

The first group received the compound naphthalene-1,3,7-trisulfonic acid (N3SA), a nanomolar-potent stabilizer of NAPE-PLD known to bind the enzyme’s internal dimeric channel. The second group was treated with the compound naphthalene-1,3-disulfonic acid, 7-hydroxy (N2SA7), a structural analogue of N3SA in which the sulfonic acid group at position 7 is substituted with a hydroxyl group (–OH). The last group received Suramin, a drug molecule containing naphthalene-1,3,5-trisulfonic acid as the active moiety. The structure of N3SA and the selected analogues are shown in Figure 1 (Suramin, R = 8-[[4-methyl-3-[[3-[[3-[[2-methyl-5-[(4,6,8-trisulfonaphthalen-1-yl)carbamoyl]phenyl]carbamoyl]phenyl]carbamoylamino]benzoyl]amino]benzoyl]).

Within each group, three animals were designated for the evaluation of cerebral microcirculation, specifically the analysis of pial arteriolar vasomotion, while one animal per group was used to assess peripheral microcirculatory dynamics, focusing on the arterioles of the femoral muscle.

### 4.2. Operative Procedure to Assess the Arteriolar Diameter Changes

For in vivo analysis of cerebral and peripheral microcirculation, animals were anesthetized with α-chloralose (60 mg/kg, intraperitoneally), a protocol chosen to ensure deep anesthesia while preserving autonomic cardiovascular regulation. Once the animal has been anesthetized, a 3 Fr polyurethane catheter, approximately 25 mm in length with an inner diameter of 2.5 mm, was positioned within the trachea to enable mechanical ventilation, and the right femoral vein was catheterized to allow intravenous administration of the test compounds [28]. Animals were placed on a heating stereotaxic frame to maintain the body temperature constant at 37.0 ± 0.5 °C.

A closed cranial window, measuring approximately 4 × 5 mm, was surgically prepared over the parietal cortex, located 1.5 mm posterior to the bregma and 3 mm lateral to the midline. The exposed pial surface was continuously perfused with artificial cerebrospinal fluid (aCSF) to maintain physiological conditions. At the same time, the left femoral muscle was exposed and perfused with a warmed physiological solution to allow visualization of peripheral microvascular networks.

Fluorescein isothiocyanate (FITC) conjugated dextran (MW 70 kDa) was administered intravenously to observe, in vivo by a fluorescence microscopy technique (Leitz Orthoplan, ERNST LEITZ WETZLAR GMBH 6330 Wetzlar, Wetzlar, Germany), fitted with long-distance objectives and eyepiece and a filter block (Ploemopak Leitz, ERNST LEITZ WETZLAR GMBH 6330 Wetzlar, Wetzlar, Germany), the pial and femoral microvasculature under baseline conditions and at 10, 20, 30, 40, and 50 min after drug administration, enabling real-time assessment of arteriolar tone changes.

The diameter and length of the vessels were quantified offline using a dedicated computer-assisted imaging analysis system (MIP Image, CNR, Institute of Clinical Physiology, Pisa, Italy) applied to continuously acquired videotaped recordings. All measurements were independently performed by two blind operators to ensure objectivity and reproducibility.

Arteriolar network maps were reconstructed by extracting stop-frame images from the video recordings. Within each map, pial arterioles were classified according to a centripetal hierarchical scheme based on the modified Strahler method, which accounts for vessel diameter as a primary criterion for branching order [29].

Rhythmic arteriolar diameter changes were analyzed using a computer-assisted power spectrum. The data shown was collected in order 2 pial arterioles, which have been identified as the most responsive to regulatory mechanisms and the most numerous in the pial microcirculation in the SHRs [30].

For an accurate comparison at the peripheral level and because a classification of rat femoral muscle arterioles is not available, the diameter rhythmic changes were assessed in a femoral arteriole whose baseline diameter fell within the range characteristic of second-order cerebral arterioles (mean diameter: 26.2 ± 0.9 µm).

The rhythmic arteriolar diameter changes were analyzed using a computer-assisted power spectrum method based on the Generalized Short-Time Fourier Transform (GSTFT), as previously described [17,31,32]. This approach enabled time–frequency domain analysis of microvascular tone by focusing on the specific time point at which each compound exerted its maximal vasomotor effect. Spectral decomposition allowed for the identification and quantification of distinct frequency components involved in the regulation of arteriolar tone.

Six physiological frequency bands were characterized: ultra-low frequency (ULF, 0.005–0.0095 Hz), reflecting nitric oxide (NO)-independent endothelial activity; very low frequency (VLF, 0.0095–0.02 Hz), associated with NO-dependent endothelial modulation; intermediate low frequency (ILF, 0.02–0.06 Hz), indicative of neurogenic control; low frequency (LF, 0.06–0.2 Hz), corresponding to myogenic activity; high frequency (HF, 0.2–2.0 Hz), linked to respiratory-related oscillations; and very high frequency (VHF, 2.5–4.5 Hz), representing cardiac activity. The power spectrum associated with each of the six frequency components is expressed as a percentage of the total spectral power, such that the sum of all components equals 100%. This normalization allows for the relative contribution of each physiological mechanism to be quantitatively compared.

The diameter values were obtained on 20 min tracings [17]. Moreover, in pial arterioles, the spectral analysis was performed in the time interval between 20 and 40 min after drug administration, when the vasodilation was evident. As we are interested in the mechanisms underlying vasodilation induced by the tested substance in femoral muscle arterioles, we carried out the spectral analysis in the time intervals that included an evident vasodilation effect.

### 4.3. Statistical Analysis

Variations in the diameter of pial arterioles, as well as in the corresponding frequency components, were statistically analyzed using two-way repeated-measures analysis of variance (ANOVA), in order to assess both intra- and inter-group effects over time. Bonferroni test applied for “post hoc” comparisons when the ANOVA revealed a statistically significant effect (*p* < 0.05). The statistical analysis was performed by SPSS 14.0 statistical package (IBM Italia, Segrate, MI, Italy).

## 5. Conclusions

Notably, our study combines intravital imaging with power spectral analysis to investigate structure–activity relationships at the basis of vascular responses in the modulation of the target NAPE-PLD. We demonstrate that they have a direct impact on local microvascular oscillatory mechanisms. The results highlight the sensibility of our methodology to discriminate even small chemical differences among antihypertensive agents targeting the novel systemic membrane target. Our approach can finely describe in vivo the arteriolar tone regulation during the time upon compound administration.

Our findings consolidate the role of NAPE-PLD of the endocannabinoid system as a molecular effector of thiazide antihypertensive action. They give a vision on how the modulation of this target affects the systemic microcirculation, from its endothelial, muscle, and neuronal tissues to the cardiac and respiratory responses. They also provide a promising strategy to evaluate small chemical modifications that can reduce the adverse effects commonly associated with traditional thiazide diuretics (e.g., hyponatremia) [19] as well as novel agents (e.g., N3SA) during preclinical research. Among these, those modulating neurovascular dysfunctions and metabolic comorbidities associated with NAPE-PLD activity [11,12]. Exploring the effects of N3SA analogues using our approach in other preclinical models of hypertension and diverse pathological conditions might help to investigate how NAPE-PLD manages to balance the vascular and neural components.

## Data Availability

The original contributions presented in this study are included in the article/Supplementary Materials. Further inquiries can be directed to the corresponding author.

## Supplementary Materials

The following supporting information can be downloaded at: https://www.mdpi.com/article/10.3390/ijms27021006/s1.
