# Supplementary figures and images for: Effects of N3SA Analogues on Cerebral and Peripheral Arteriolar Vasomotion in Spontaneously Hypertensive Rats
Source: Int J Mol Sci. 2026 Jan 20;27(2):1006. doi: 10.3390/ijms27021006 (PMC12842546; doi:10.3390/ijms27021006)

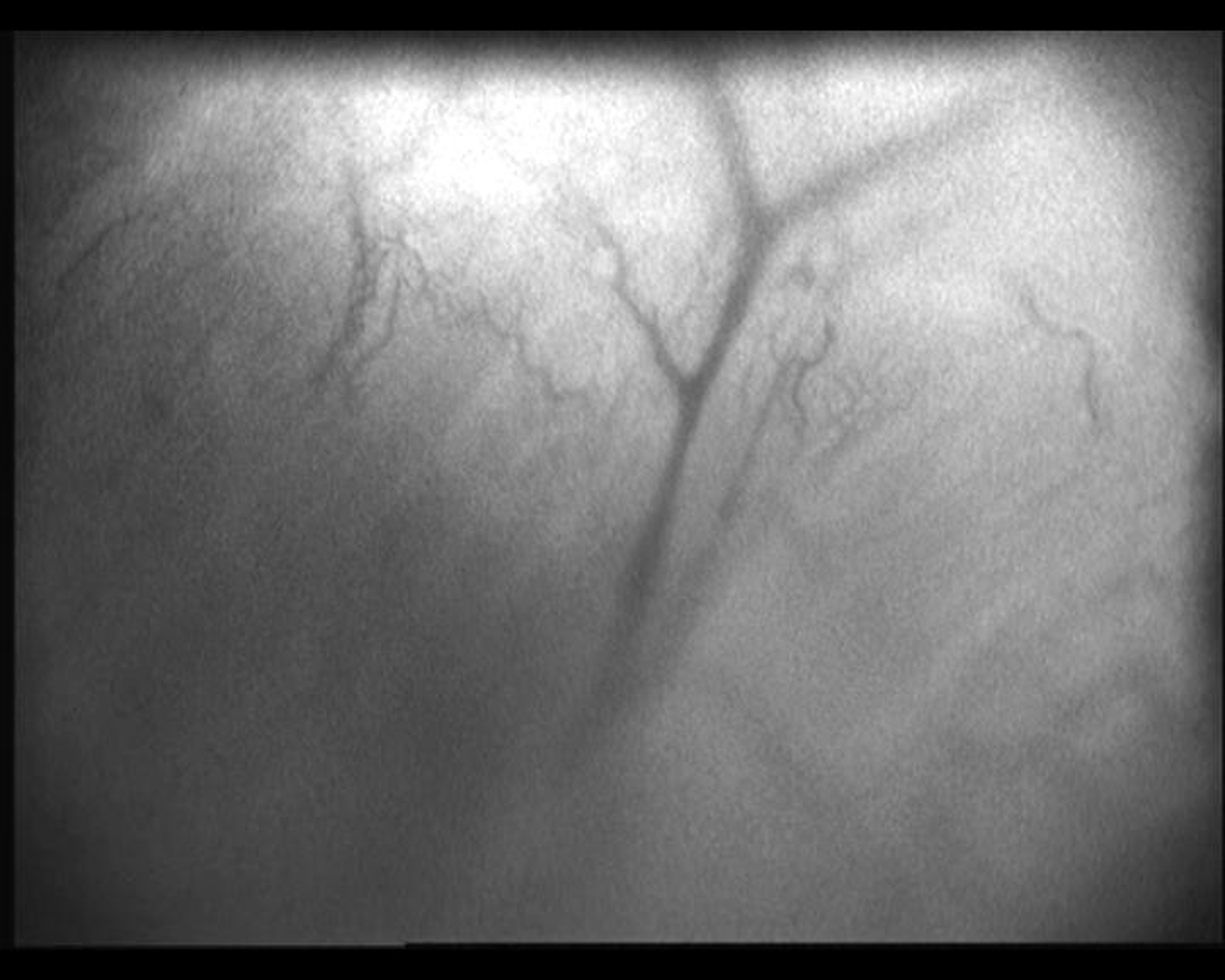

Supplement: Supplementary file 1 [file ijms-27-01006-s001.zip › original frames for femoral arterioles/10 min.tif]

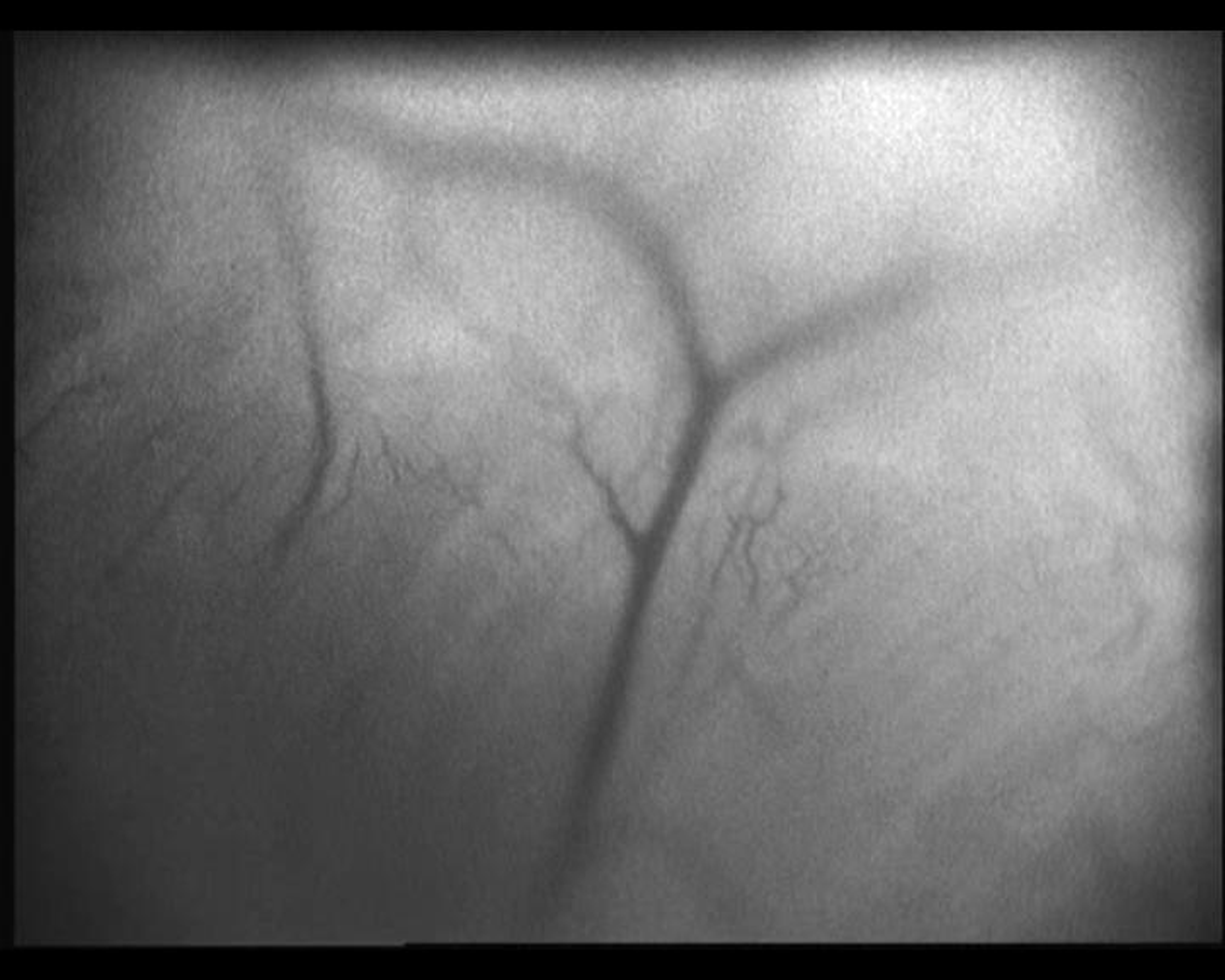

Supplement: Supplementary file 1 [file ijms-27-01006-s001.zip › original frames for femoral arterioles/20 min.tif]

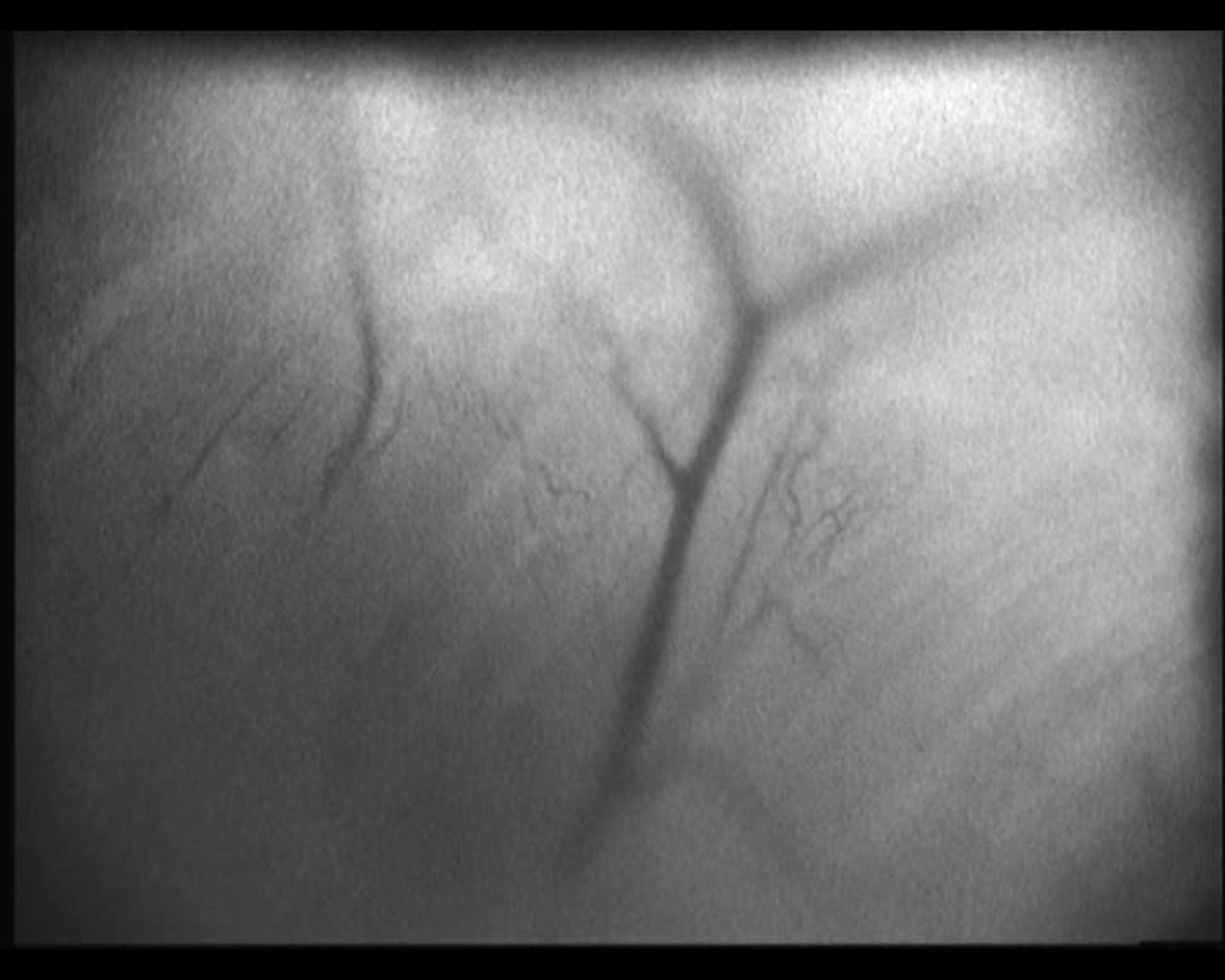

Supplement: Supplementary file 1 [file ijms-27-01006-s001.zip › original frames for femoral arterioles/30 min.tif]

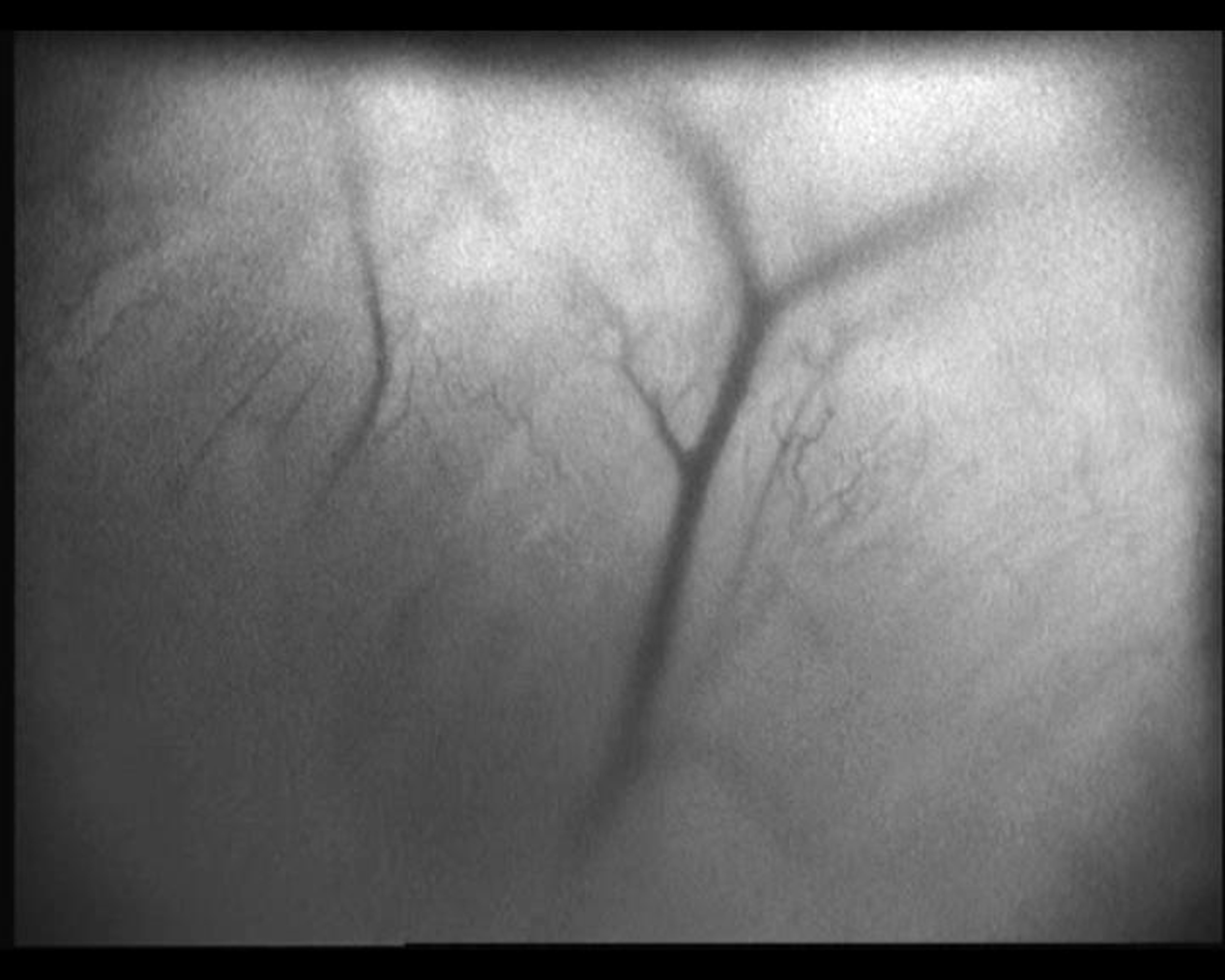

Supplement: Supplementary file 1 [file ijms-27-01006-s001.zip › original frames for femoral arterioles/40 min.tif]

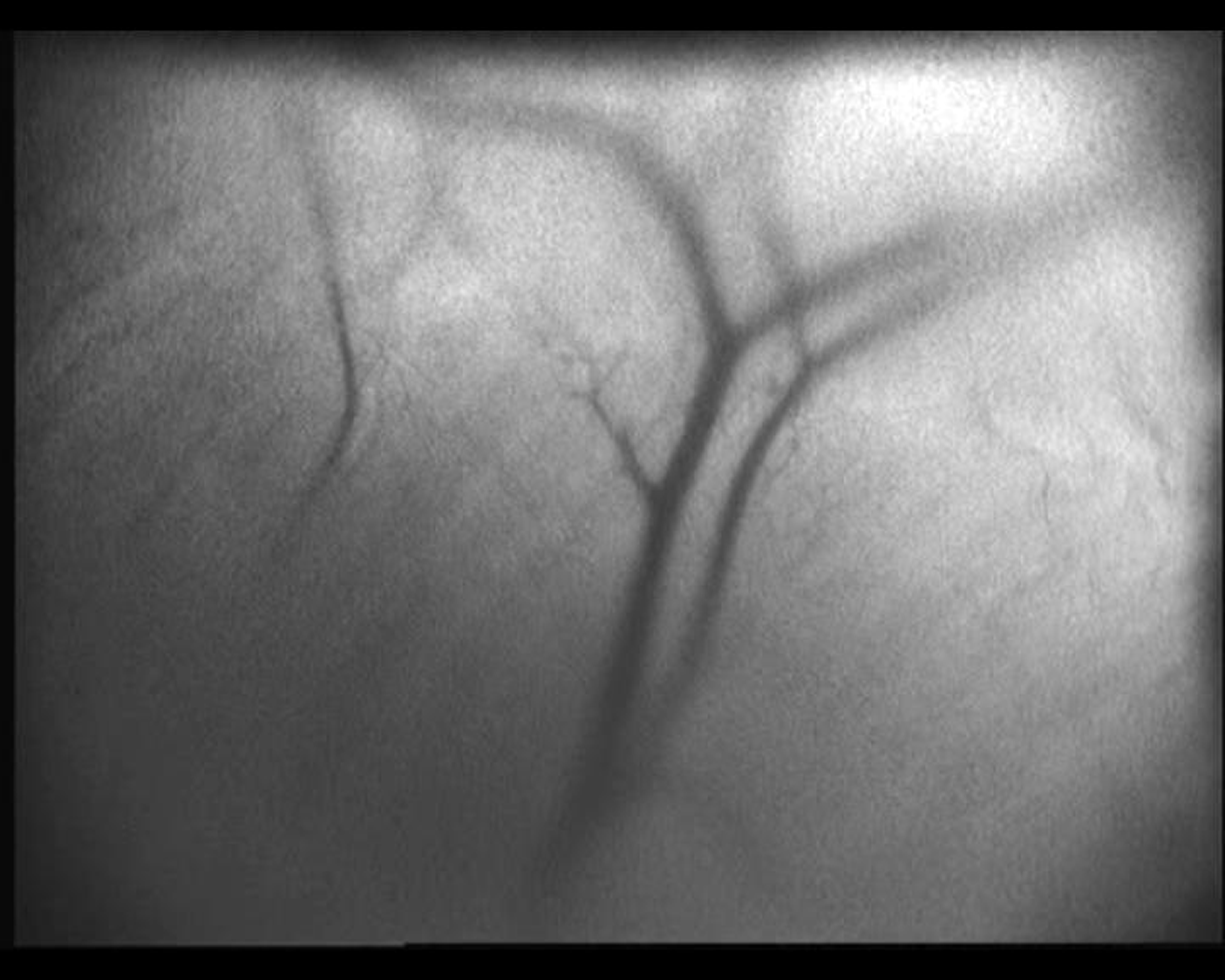

Supplement: Supplementary file 1 [file ijms-27-01006-s001.zip › original frames for femoral arterioles/50 min.tif]

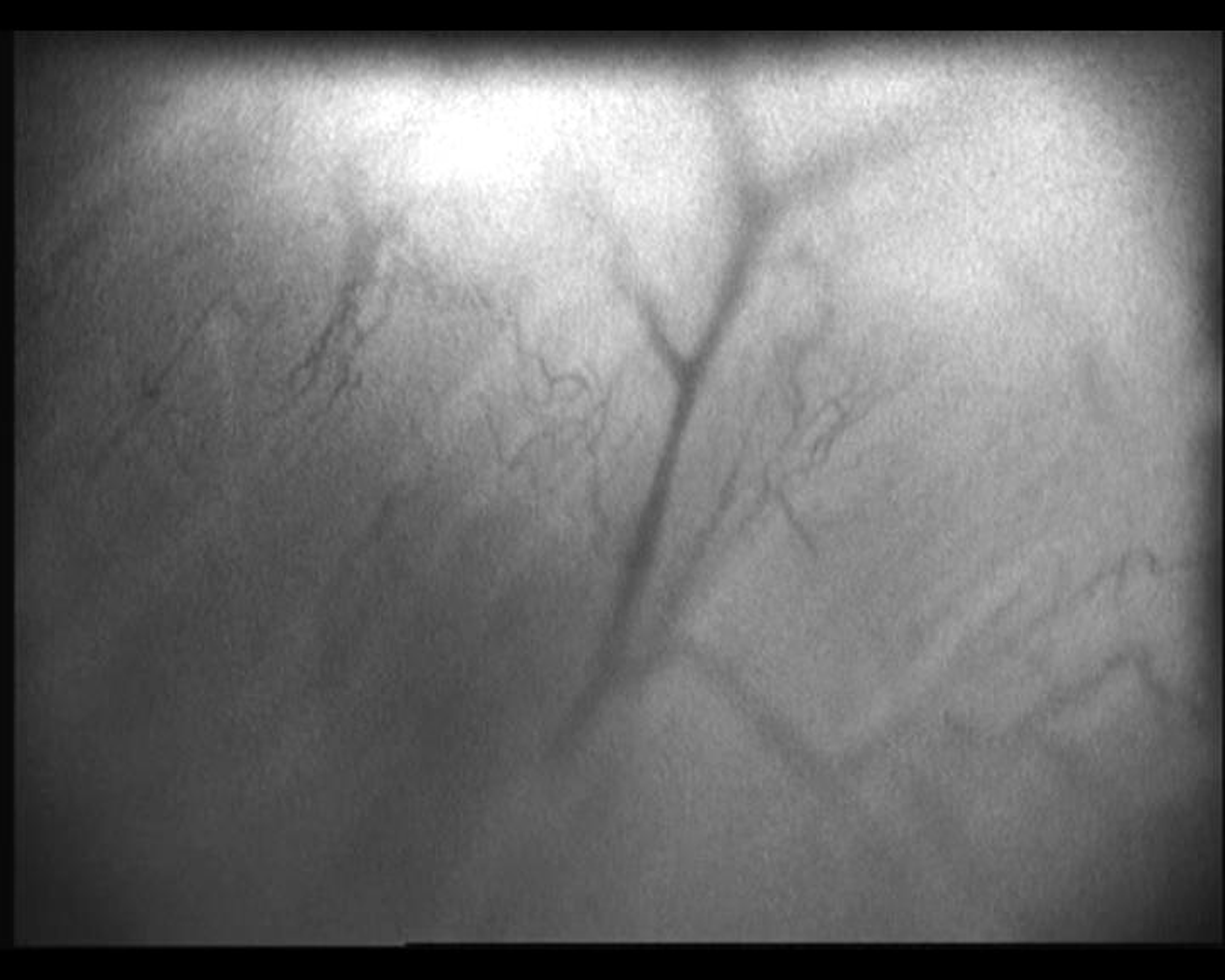

Supplement: Supplementary file 1 [file ijms-27-01006-s001.zip › original frames for femoral arterioles/Basal.tif]

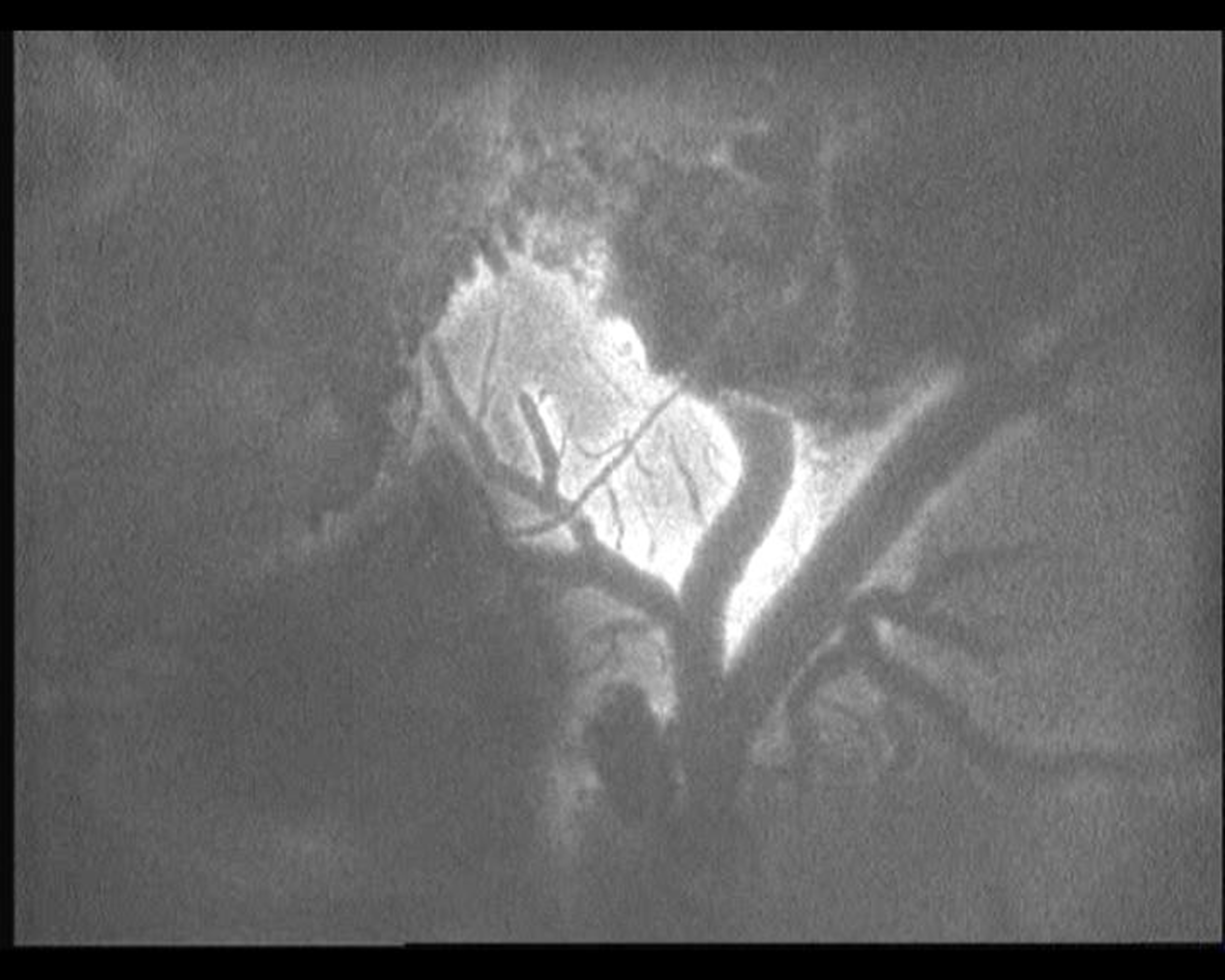

Supplement: Supplementary file 1 [file ijms-27-01006-s001.zip › original frames for pial arterioles/10 min copia.jpg]

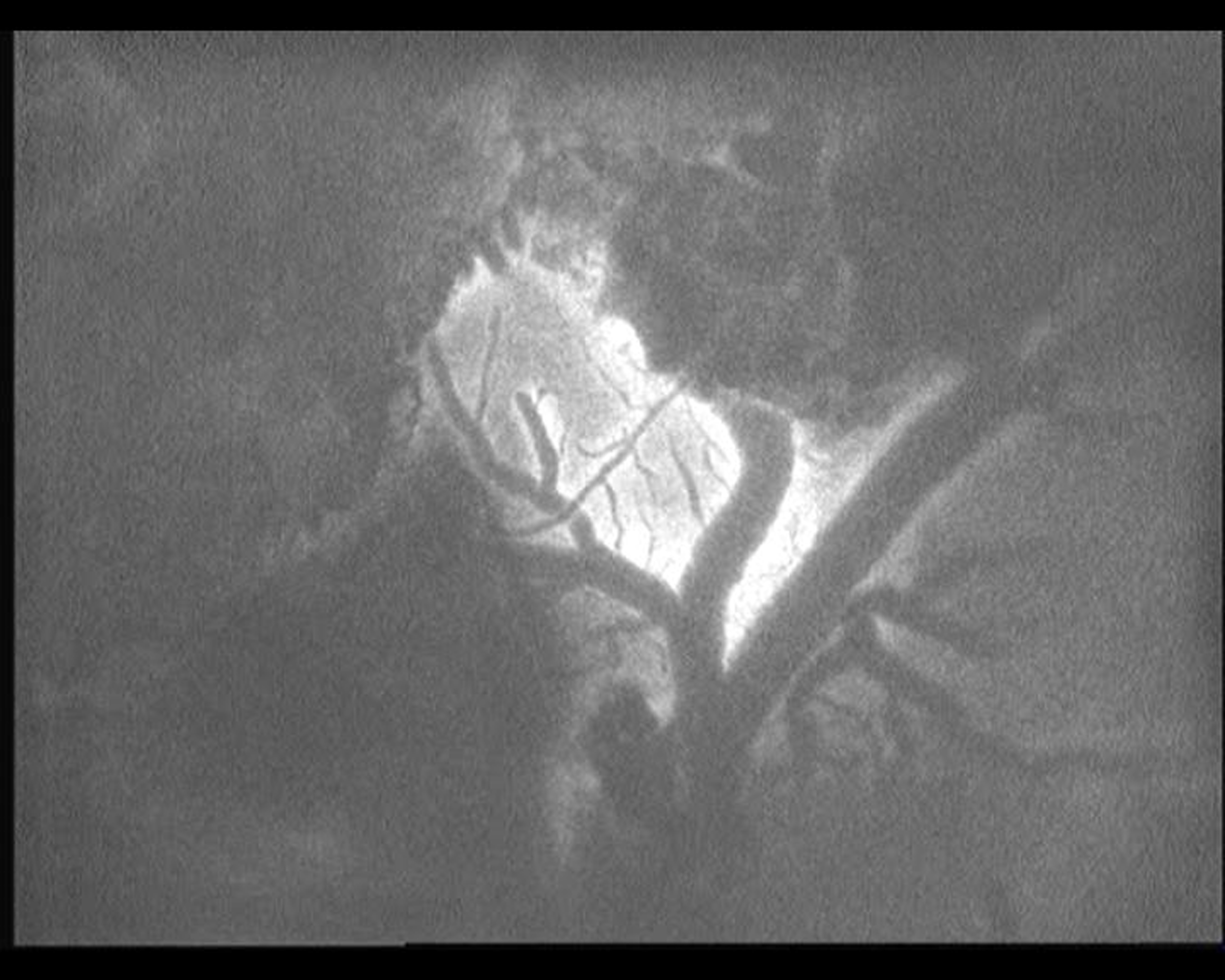

Supplement: Supplementary file 1 [file ijms-27-01006-s001.zip › original frames for pial arterioles/20 min copia.jpg]

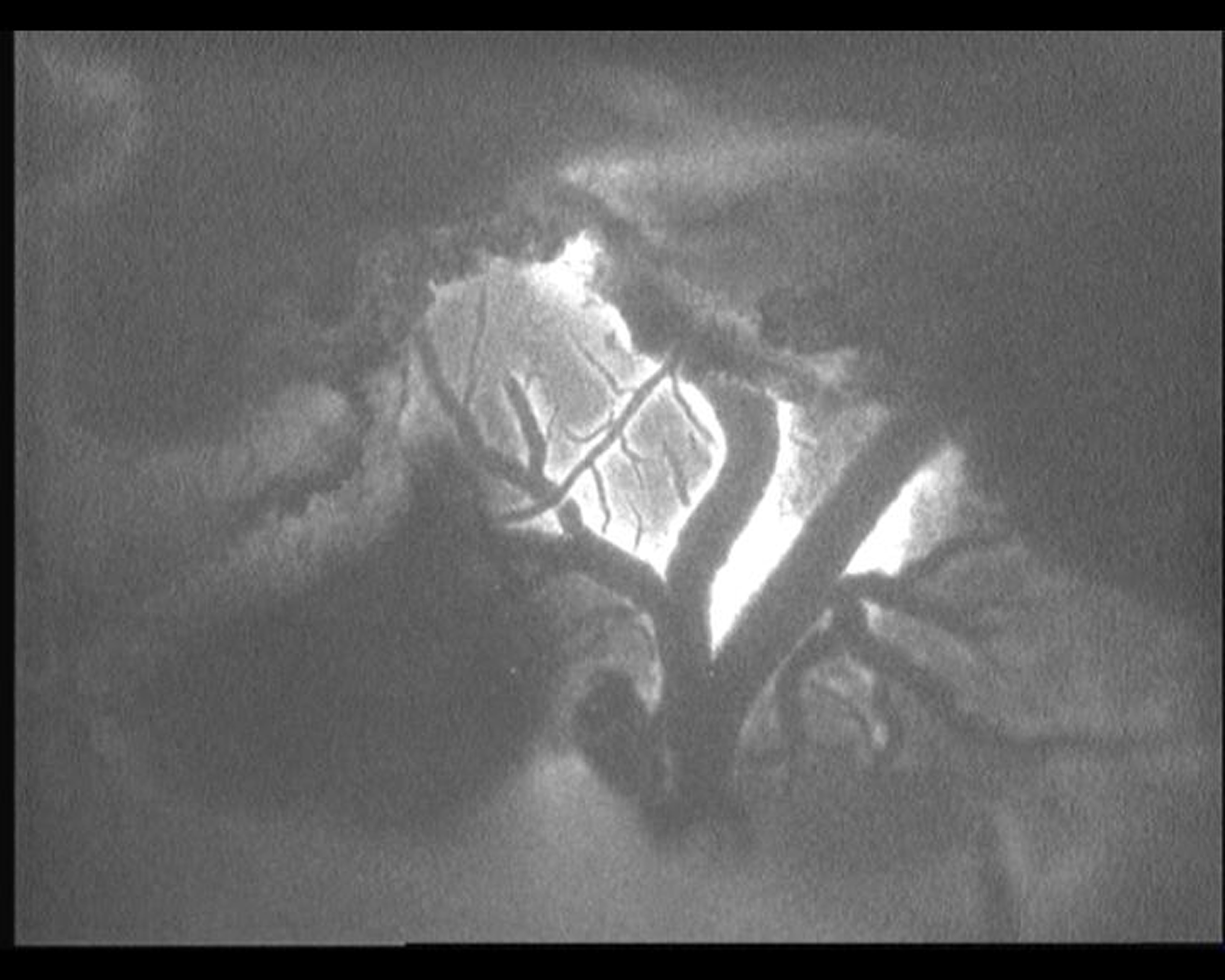

Supplement: Supplementary file 1 [file ijms-27-01006-s001.zip › original frames for pial arterioles/30 min copia.jpg]

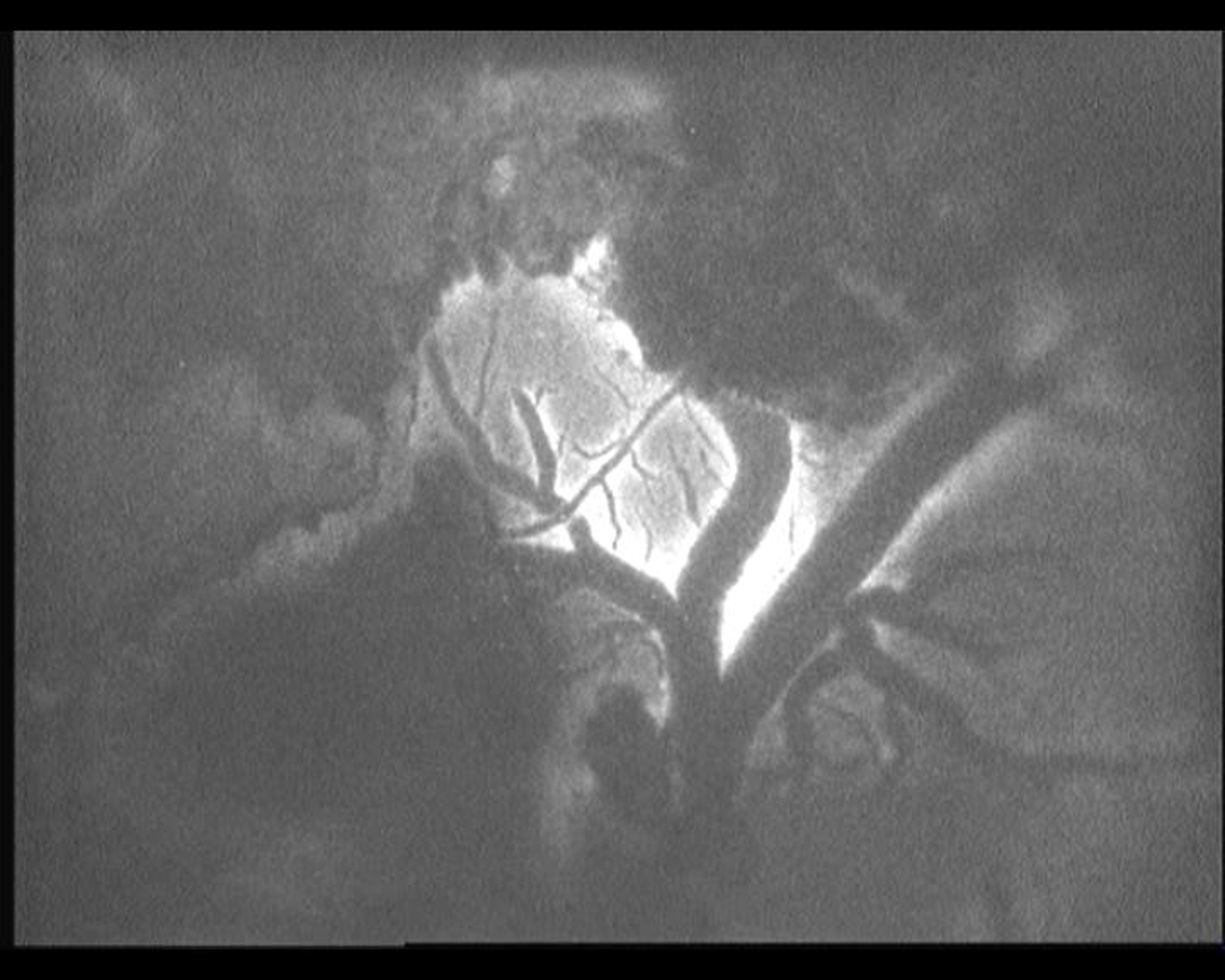

Supplement: Supplementary file 1 [file ijms-27-01006-s001.zip › original frames for pial arterioles/40 min copia.jpg]

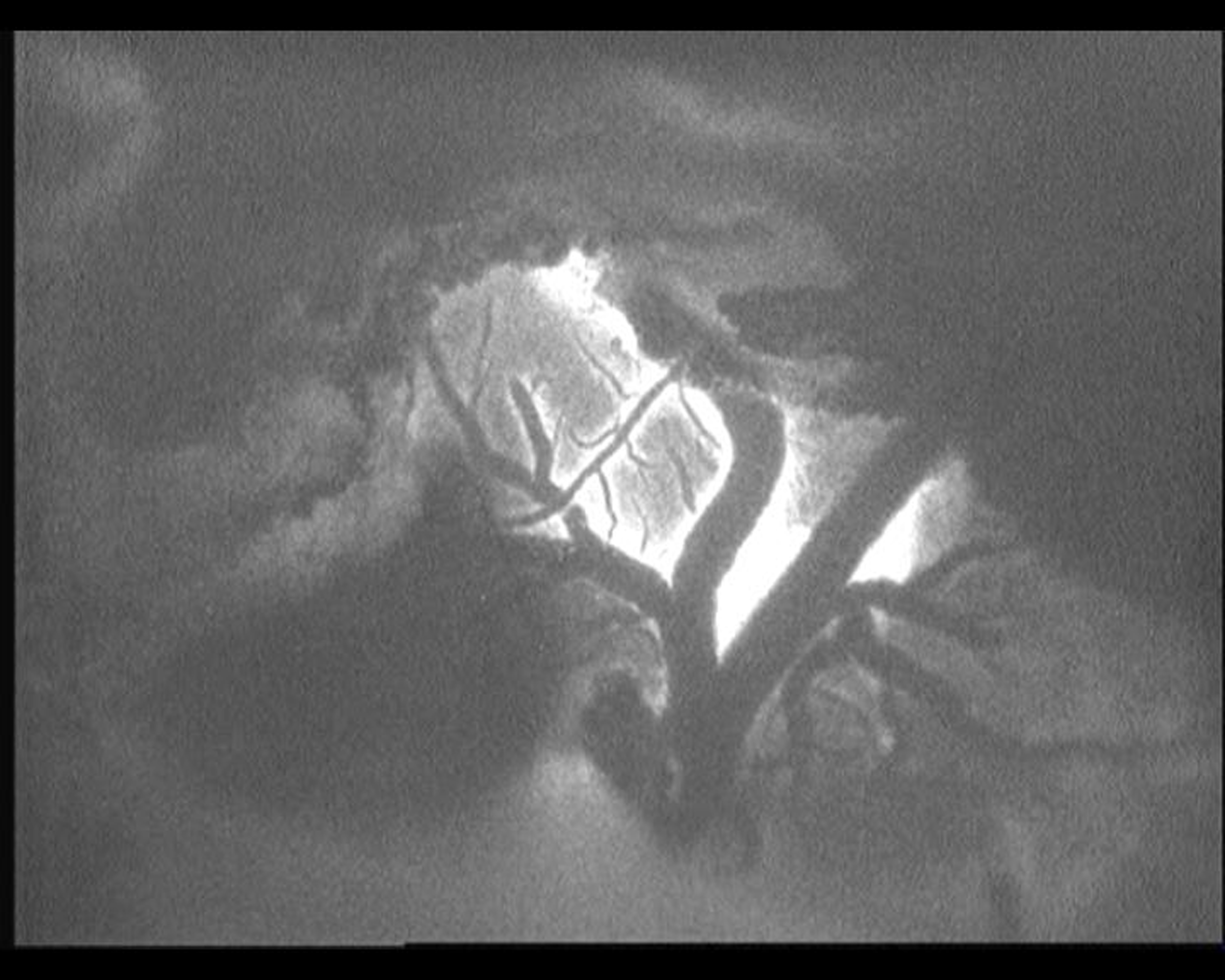

Supplement: Supplementary file 1 [file ijms-27-01006-s001.zip › original frames for pial arterioles/50 min copia.jpg]

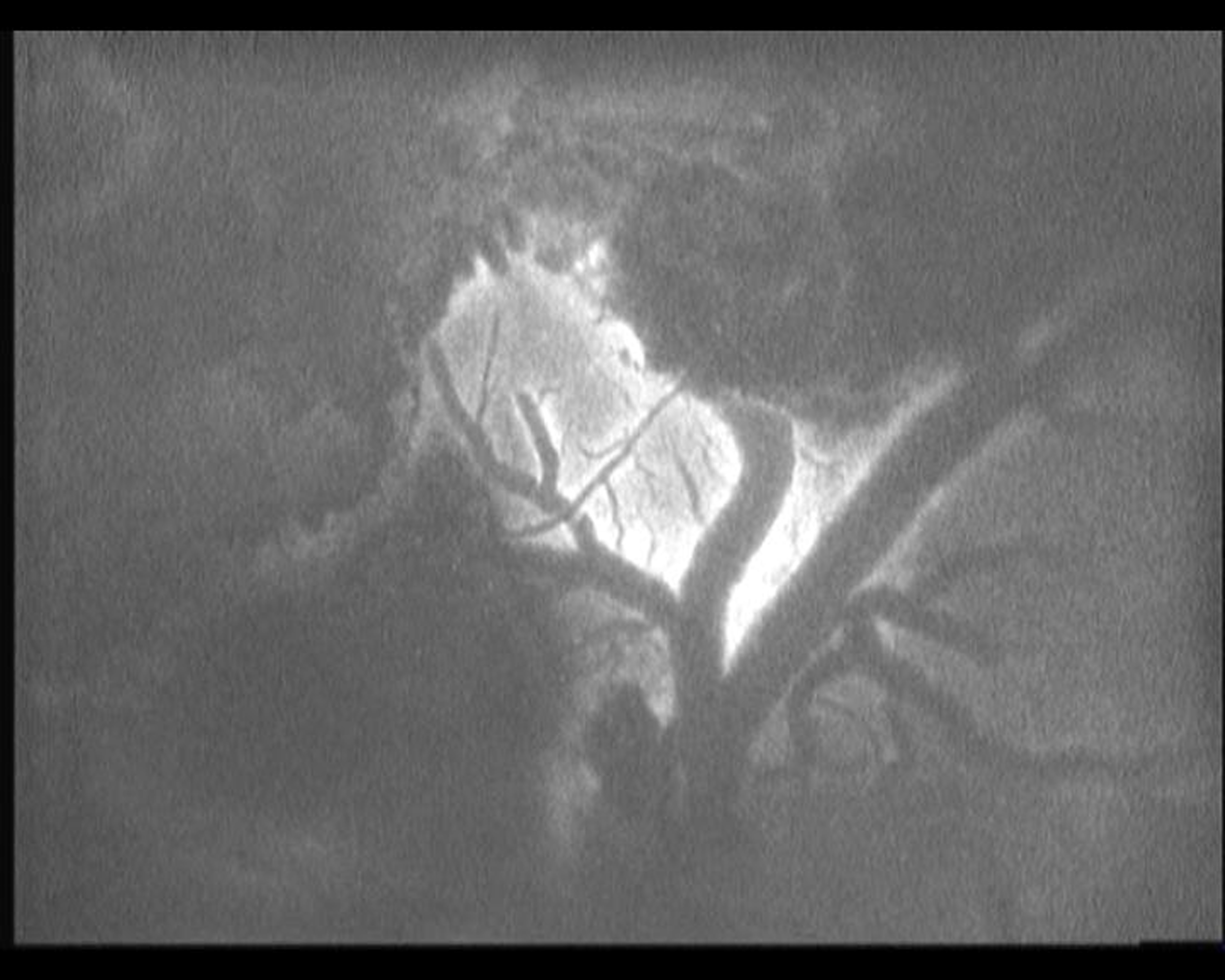

Supplement: Supplementary file 1 [file ijms-27-01006-s001.zip › original frames for pial arterioles/Basal copia.jpg]
